# The Effect of TiN and DLC Anti-Wear Coatings on the Tribofilm Formation and Frictional Heat Phenomena in Coated Metals vs. WC-Co

**DOI:** 10.3390/ma14123342

**Published:** 2021-06-17

**Authors:** Magdalena Łępicka, Yurii Tsybrii, Daniel Kiejko, Karol Golak

**Affiliations:** Institute of Mechanical Engineering, Faculty of Mechanical Engineering, Bialystok University of Technology, Wiejska 45C, 15-532 Bialystok, Poland; y.tsybrii@pb.edu.pl (Y.T.); dkiejko@gmail.com (D.K.); k.golak@pb.edu.pl (K.G.)

**Keywords:** wear, frictional heat, surface modification

## Abstract

The aim of this work was to study the effect of anti-wear coatings on the selected frictional phenomena, i.a., frictional heating and tribofilm formation, of model tribological pairs. For this purpose, three popular metallic substrate materials were selected: AISI 316L and AISI 440B stainless steels, as well as Ti6Al4V two-phase titanium alloy. The substrates were tested in the dry sliding conditions in three states: uncoated, as well as titanium nitride (TiN) or diamond-like-carbon (DLC) coated. According to the results provided, under applied frictional conditions TiN coating, even if it is worn off the sample surface, contributes to excessive frictional heating of a tribological pair by altering the tribofilm formation. The analysis also showed that in some tribological pairs, rapid temperature alteration of a counter sample can be used to approximate the sliding distance after which the TiN coating becomes worn off. On the contrary, in all pairs tested, the DLC film became locally damaged, but it sustained its antifriction properties, contributing to low coefficients of friction (COFs) and the lowest frictional temperatures observed.

## 1. Introduction

When designing a surface-modified metallic frictional pair, it is crucial to be able to assess the longevity of the protective film. As the previous studies showed, it is possible to prognose, to some extent, the service life of a coating deposited on a metallic substrate [1,2]. According to the numerous authors, the ratios of hardness to Young’s modulus of the film, e.g., strain to failure [1] or plasticity index [2], as well as the mismatch between Young’s modulus of the film and the substrate [3], substantially affect the service life of a thin coating. However, the abovementioned methods cannot be used to monitor the current state of a tribopair. For example, strain to failure or plasticity indices are not applicable in terms of spotting the transition between stable and catastrophic wear of the protective film. When stable wear of a frictional pair is present, the friction couple can operate without interference. Nevertheless, progressing damage of the film and release of hard abrasive microparticles can lead to excessive wear. Therefore, numerous efforts are being undertaken to improve research methods to the extent where it will be possible to spot the progression in wear and, most importantly, the impending peeling off of the coating and subsequent exposure of the substrate material.

While some researchers have shown that the failure of an anti-wear film can be prognosed based on dynamics of coefficient of friction (COF) [4,5,6], there are tribopairs in which this method will not succeed. It was presented that the value of COF depends not only on the contact surface, environment [7], or the lubricating conditions. The instability of lubricating conditions, tribofilms formation, as well as texturization of the wear track contributes to the oscillations in COF [8]. Therefore, in some cases, changes in COF dynamics may not be sufficient to spot impending wear of the coating.

Due to that, in wear testing, electrical contact resistance (ECR) measurements are being proposed [9]. In dry conditions, the in situ ECR monitoring can be utilized to spot the failure of a coating, as well as to determine whether a metallic substrate material is in direct contact with the counter body [9]. However, this method also has its shortcomings. First, the triboelectric effects depend greatly on the mating materials, test conditions, as well as wear of the tribological pair [10]. For example, Wei et al. [11] presented that the applicability of ECR monitoring is limited by the dominant wear mode of the frictional pair. According to the authors, the occurrence of the worn particles of the film in the friction zone may lead to a lack of response in ECR dynamics. Therefore, no progress in the failure of the protective film can be observed. Moreover, Grandin and Wiklund [12] showed that the progression in sample wear which increases the contact area may cause a decrease in ECR. Therefore, based on ECR signals only, incorrect conclusions can be drawn.

In some tribological pairs, the temperature response is monitored simultaneously with COF oscillations. Frictional heating of a tribological pair is a result of the friction and wear phenomena that occur in the contact zone. There are numerous reports on methods for measuring temperature in tribological contact, as well as the influence of the surface asperities, phase transitions, or frictional film formation on the thermal response of a tribopair [7,13]. For example, contact temperature measurements are often done when tribologically testing brake pads [14,15]. The results, e.g., oscillations of temperature in the contact region, as well as analyses of the heat flux, are being used to discuss the friction and wear phenomena [14,15]. Moreover, temperature sensing can be used to study the contact area or the heat-transfer capability of a developed material. For example, in the work by Chey et al. [16], a model for estimating real frictional contact area from the interface temperature was presented. Straffelini et al. [17] empirically measured and then modeled the evolution of the pin and disc temperature during friction. Ming et al. [18] studied the heat-transfer capability of Ti6Al4V alloy in frictional conditions. However, the literature lacks model studies on the effect of surface coating on the thermal response of metallic materials, as well as patterns for frictional film formation after the failure of the coating occurs.

Surface modification is nowadays one of the most popular methods for enhancing the service life of modern mechanical systems. The routinely applied surface modification methods include but are not limited to physical vapor deposition, chemical vapor deposition, thermochemical treatment, etc. [19,20,21,22]. Among applied coatings, titanium nitride (TiN) and diamond-like-carbon (DLC) are routinely used to improve tribological performance of metallic materials. Their exceptionally good anti-wear performance, as well as chemical inertness [23,24], were discussed in numerous scientific reports. However, in the literature in the field, works devoted to friction, wear and heat phenomena of surface-modified model metallic materials working in dry conditions are still scarce. In most cases, the effect of the elevated temperature on the stability of the coating [25,26] or the frictional response of coated metals [19,27,28] is presented. Little or no attention is being paid to the influence of the coating on the friction-induced thermal behavior of a frictional pair, as well as tribofilm formation. In this work, a comparison of tribological and thermal responses of model surface-modified metallic materials is presented. The applicability of temperature sensing in coated metals is discussed along with patterns of frictional film formation.

## 2. Materials and Methods

### 2.1. Materials

The friction studies were conducted for three model metallic materials: AISI 316L austenitic stainless steel, AISI 440B martensitic stainless steel, and Ti6Al4V two-phase titanium alloy. The considered alloys were selected as model materials for the frictional test due to their extreme popularity in the machine industry. In Table 1, the chemical composition of 316L stainless steel is given. Products made of 316L steel are characterized by resistance to elevated temperatures and high plasticity [29]. Therefore, this steel is widely used in medicine, including the production of surgical instruments, as well as in the chemical, food, mining, and petrochemical industries. On the other hand, the second selected substrate material, titanium alloy Ti6Al4V, found its applications in the aviation, automotive, chemical and biomedical industry. The material is often used in the mentioned branches of industry because of its low density, high biocompatibility, as well as the high strength-to-weight ratio [30]. Due to the tendency to form and self-reproduce an oxide protective layer on its surface in ambient conditions, this alloy also exhibits good corrosion resistance. Table 2 presents the chemical composition of the Ti6Al4V titanium alloy. The third selected substrate material, AISI 440B stainless steel, is one of the hardest of martensitic stainless steels [31]. Due to that, it is often used in a variety of industrial applications such as molds, tools, structural parts of automotive and biomedical devices. Its high wear and moderate corrosion resistance are attributed to its high carbon and chromium content (Table 3). On the other hand, as the counter body for the frictional tests, Ø6 mm sintered tungsten carbide (WC-Co) balls were selected. Typically, in model frictional tests, balls of AISI 52100 bearing steel are recommended [32]. However, in preliminary studies, it occurred that AISI 52100 bearing steel is not wear-resistant enough, as it was severely worn in contact with coated materials. Then, instead of maintaining non-conformal contact with the tested material, the counter body would tend to flatten. Therefore, as counter material, a hard sintered carbide (WC-Co) was chosen.

As can be seen, model materials of various properties were selected for the study. In tribology, it is well known that hardness is one of the main factors which determines the wear performance of metals. In Table 4, the hardness of all substrate materials is given. As can be seen, considering only the hardness of the substrates, a variety of tribological responses can be expected, as a difference in Vickers hardness between model metallic materials is significant. From the selected materials, test samples with a diameter of 25 mm and a height of 6 mm were fabricated. The samples which were used in the study were subjected to mechanical grinding, and then hand polished on a microfiber polishing pad. After that, some specimens were subjected to surface modification with two coatings.

**Figure 1:**
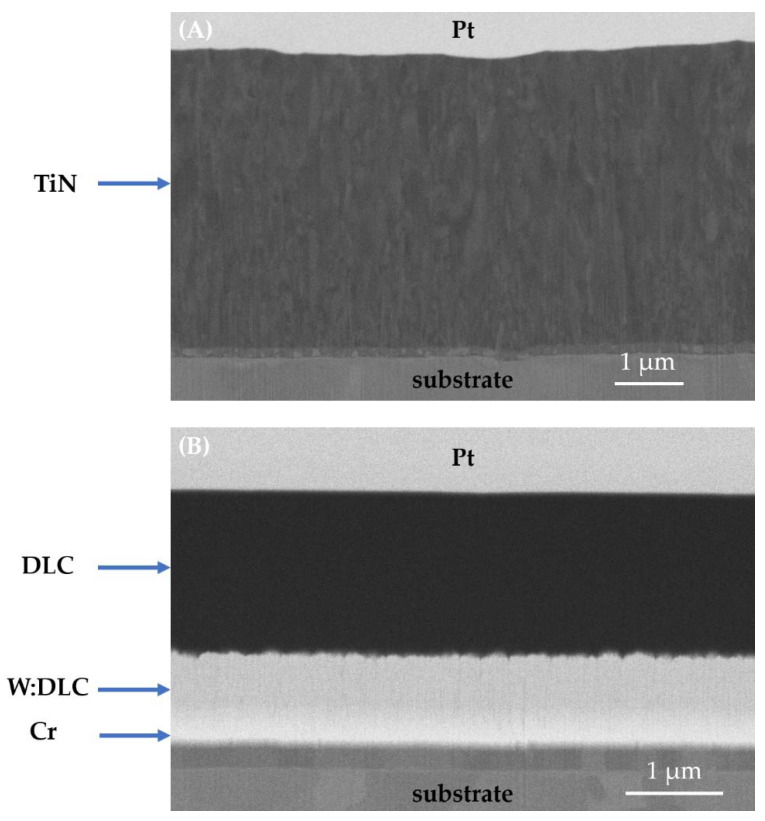
FIB-SEM cross sections of: (**A**) titanium nitride, (**B**) hybrid DLC coating. Pt is a protective layer deposited in situ during preparation of a cross section.

To ensure repeatability of the conducted experiments, as well as consistency in quality of the analyzed samples, two commercial coatings were selected: monolayer titanium nitride (TiN) and hybrid diamond-like-carbon (DLC). Both films were selected as model materials due to their high popularity in machine engineering. Before the thin film deposition, the substrates were thoroughly cleaned: degreased in an alkaline bath, rinsed with de-ionized water, and dried in a vacuum. The ca. 4.2 µm thick TiN coating (Figure 1A) was prepared by the cathodic arc deposition technique (CAE-PVD) from the Ti target in Ar + N_2_ mixture. On the contrary, for the hybrid DLC coating deposition, magnetron sputtering (MS), as well as plasma-assisted chemical vapor deposition (PACVD), were used. Unbalanced magnetron sputtering from Cr target was utilized to prepare a thin, adhesive chromium layer. Then, a support W:DLC sublayer was obtained in an acetylene gas environment by the MS + PACVD technique. The top layer, pure DLC, obtained by PACVD from acetylene, was used. Therefore, a multilayer stacked hybrid DLC coating of a total thickness of ca. 2.8 µm was obtained (Figure 1B). A summary of all samples tested is given in Table 1. The total number of samples used in the study was 45. In Table 1, the average surface roughness (Ra) of the samples, measured with the use of a contact profilometer (Hommel-Etamic T1000, Jenoptic, Jena, Germany), is included. The Ra measurements were taken in accordance with ISO 4288:2011.

### 2.2. Methods

**Figure 2:**
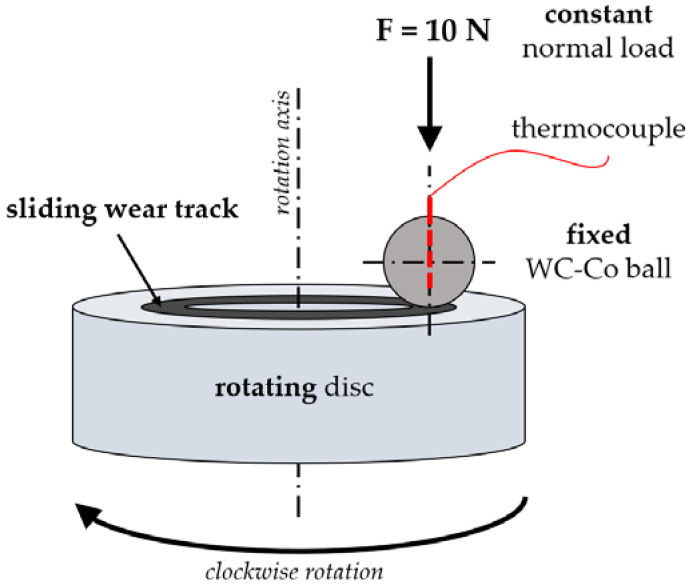
A schematic representation of the ball on disc configuration used during the sliding wear tests.

The tribological tests were done on a T-11 tribometer (Institute for Sustainable Technologies, Radom, Poland) in a ball-on-disc configuration (Figure 2). While the ball was fixed, the disc was rotating with a constant rotational speed. For each disc type (Table 5, S1–S9), 5 replications were carried out. For all samples, dry sliding conditions were maintained under the normal load of 10 N. The wear track radius was equal to 6 mm, while the sliding velocity was fixed at 0.1 m/s. In the frictional tests, the total sliding time equaled 7200 s, which corresponds to 1440 m of wear distance. The experiment was conducted at room temperature (21 °C) and ambient humidity. During the tribological tests, changes in coefficient of friction (COF, *µ*) in time were registered at a frequency of 10 Hz. While both friction force and normal load were known, it was possible to calculate COF with the use of the following formula:

*µ* = *T*/*F* (1)

where *T* is the friction force (N), and *F* is the applied normal load, kept at a constant level of 10 N.

Moreover, to quantify the wear of the tested tribopairs, the mass loss of each part was measured both before and after the tribological tests. The measurements were taken with a RADWAG laboratory scale (Radwag, Warsaw, Poland) with an accuracy of 10^−4^ g. As it occurred that mass loss of the counter samples was negligible, mass loss of discs only was included in the manuscript. The analyzed alloys differ significantly in density (ρ_316L_ = 8.000 g/cm^3^, ρ_Ti6Al4V_ = 4.429 g/cm^3^, ρ_440B_ = 7.740 g/cm^3^). Therefore, the wear-related changes in the mass of the samples were related to density. An indirect measurement of the volumetric wear of the discs was possible, with the use of the following formula:

Δ*V*_*t*_ = (*m_tp_* − *m_tt_*)/*ρ_mat_* (2)

where: Δ*V_t_* is the volumetric wear of disc (mm^3^), *m_tp_* is the mass of the disc registered prior to the wear test, *m_tt_* is the mass of the disc registered after the wear test, and *ρ_mat_* is the material density.

**Figure 3:**
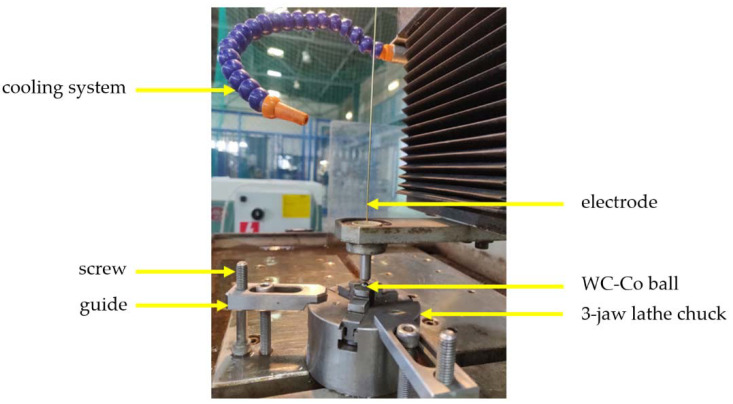
The EDM machining process of 5 mm deep blind holes (Ø1 mm) in Ø6 mm WC-Co balls.

Moreover, during the tribological tests, the friction-induced temperature alterations in the proximity to the friction zone were monitored. In WC-Co balls, with the use of electrical discharge machining (EDM), 5 mm deep blind holes with a diameter of 1 mm were made. The EDM process is an electrical discharge treatment (Figure 3). The process uses metal electrodes for drilling—in this case, Ø0.8 mm brass electrodes were selected. During EDM, the electrical discharges are passed between the electrode and the ball, separated by a dielectric layer. This way, electric current pulses are formed. The pulses act as a drilling tool [40]. A hole that reflects the shape of the electrode is formed in the machined material. Nevertheless, due to the nature of the process, the hole that is produced is slightly bigger than the diameter of the used electrode. Therefore, in effect, with the use of Ø0.8 mm electrodes, Ø1 mm holes were fabricated. Then, in the formed blind holes, in each ball used during the tests, a K-type thermocouple with a bare wire (diameter 0.5 mm, Omega Engineering Inc., Norwalk, CT, USA), with a sensing temperature range between −200 and 1250 °C, was installed. To ensure repeatability of the measurements, the gap between the electrode and the blind hole was filled with a thermally conductive Ag-based paste (AG Silver, AG TermoPasty, Sokoły, Poland). The temperature alterations at the distance of ca. 1 mm from the friction zone were sampled at a frequency of 5 Hz and low-pass filtered with a cutoff frequency of 1.5 Hz with a Graphtec GL7000/GL7-HSV data logger.

After the wear tests, samples were subjected to microscopic observations. Both the surface state of the specimens before and after the frictional tests, as well as the morphology of the wear tracks, were studied with the use of an SEM-FIB Dual Beam scanning electron microscope Scios 2 (Thermo Scientific, Waltham, MA, USA). Moreover, cross-sections of the wear tracks were prepared in situ with the use of a focused ion beam (FIB) and analyzed, i.a., with the energy dispersive X-ray sensor (EDX).

## 3. Results

**Figure 4:**
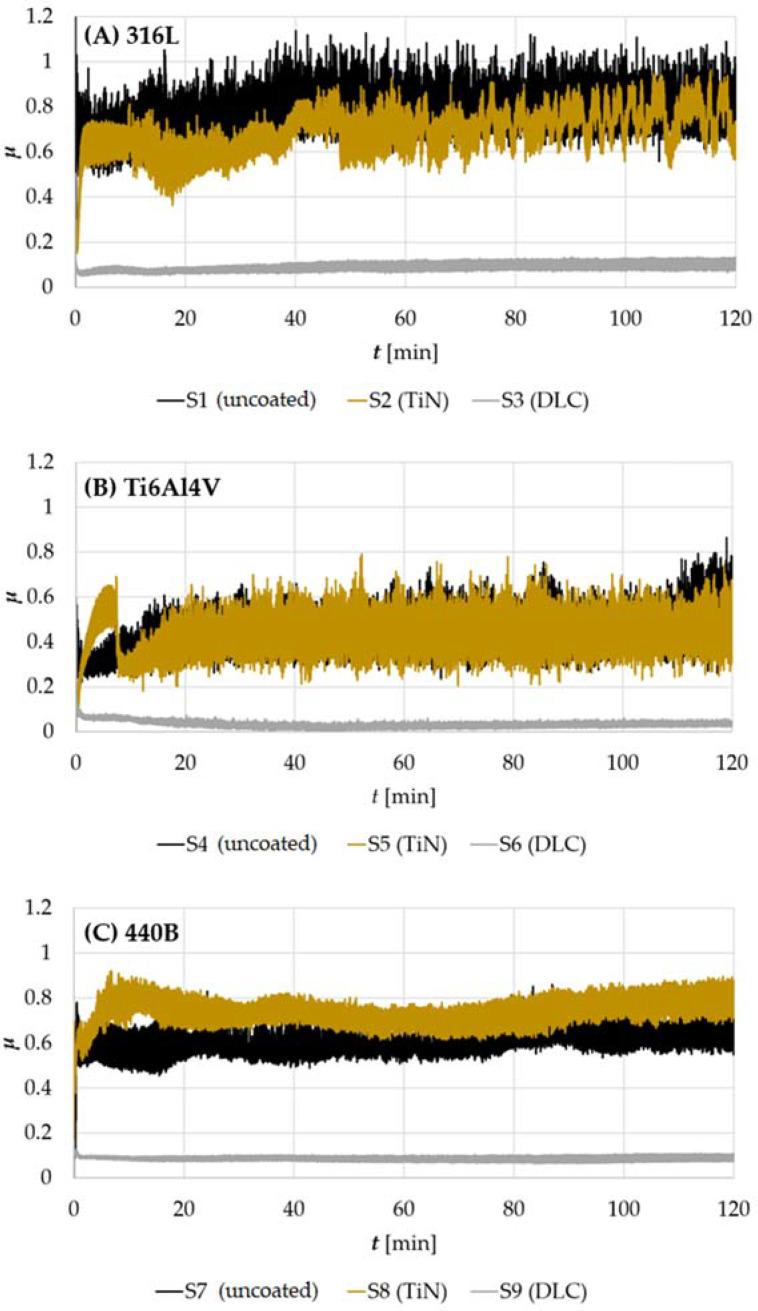
Fluctuations of COF in time, registered for samples fabricated of: (**A**) 316L austenitic stainless steel, (**B**) Ti6Al4V titanium alloy, (**C**) 440B stainless steel.

An exemplary plot of oscillations in the friction coefficient (COF), recorded for 316L samples of series S1, S2, and S3, is presented in Figure 4A. As can be seen, for the two types of specimens tested, S1 and S3, it is possible to divide the COF plots into two characteristic zones: a few minutes short running-in of the friction pair (<3 min), followed by stable wear. However, it should be emphasized that, in the stable wear phase, oscillations in *µ* are the greatest for the uncoated sample (S1). On the other hand, the smallest oscillations and the most stable frictional behavior are seen in DLC-coated steel (S3). However, the most complex frictional behavior is observed for steel modified with the TiN coating. During the first 50 min of the test, the value of the friction coefficient varies from 0.35 to 0.90. Then, the COF is set at an average level of about 0.7. This value is lower than that observed for the non-modified steel (0.8), but several times higher than that registered for the friction pair with the DLC coating (0.1).

**Figure 5:**
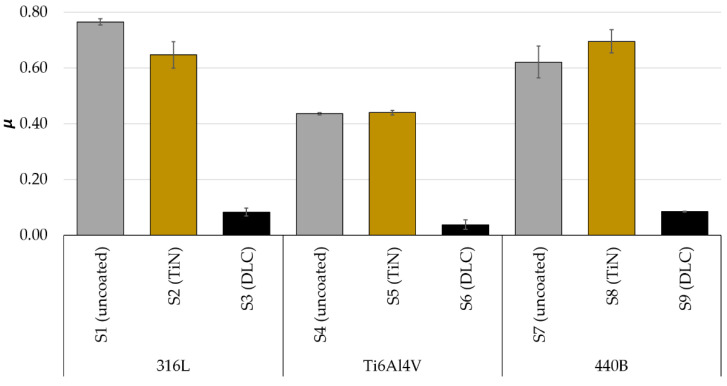
The average values of COF, registered for: 316L austenitic stainless steel, Ti6Al4V titanium alloy, and 440B stainless steel; *n* = 5 in each sample series.

A different nature of the friction coefficient fluctuations caused by friction and wear phenomena was observed for the Ti6Al4V titanium alloy (Figure 4B). First, in the case of uncoated titanium alloy, the running-in phase is longer, as stabilization of frictional behavior is seen after the first 10 min of friction. However, the value of COF observed during the stable wear range is much lower than in the case of 316L steel. Moreover, for titanium alloy modified with the TiN coating, after about 8 min from the start of the test, an increase in the amplitude of the friction coefficient in time is observed, preceded by its rapid decrease from the value of 0.65. The observed frictional response may be caused by the damage of the protective coating. This is evidenced by, among others, subsequent stabilization of the nature of the fluctuation of the friction coefficient, corresponding to the behavior of the non-surface-modified alloy. Again, the alloy modified with the DLC coating turned out to be unrivaled. The friction coefficient of the WC-Co vs. DLC Ti6Al4V tribological pair remained at an exceptionally low level, below 0.1 throughout the entire study period. The observed differences between the sample series are reflected also in the average COF values, calculated for all samples tested (Figure 5).

In Figure 4C, fluctuations in COF in time, registered for 440B stainless steel are shown. First, it can be noticed that, contrary to what was presented for 316L steel and Ti6Al4V titanium alloy, compared with the uncoated sample, the average COF increases after depositing titanium nitride coating (Figure 4C and Figure 5). The observed mean coefficient of friction of the tribopair, amounting to approx. 0.75 for TiN-modified 440B steel is higher than that recorded for the pair WC-Co vs. uncoated steel. However, as in the previous cases, the unquestionably lowest COF characterized by the smallest amplitude of oscillations can be observed for steel modified with DLC coating. 

**Figure 6:**
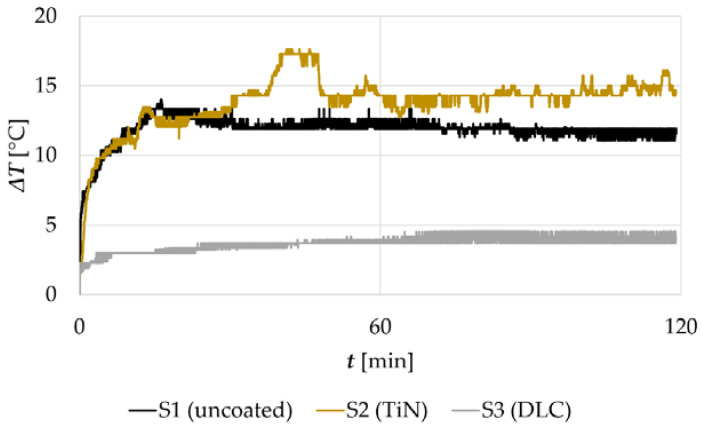
Friction-induced temperature oscillations in time, registered for sample series S1, S2, and S3. Please note that the presented temperature alterations were recorded simultaneously with data presented in Figure 2.

Simultaneously with COF, the temperature alterations of the samples were recorded. The change in temperature of each friction pair, Δ*T*, was calculated as follows:

Δ*T* = *T_a_* − *T_p_* (3)

where *T_a_* is the temperature of the friction pair recorded during the wear tests, and *T_p_* is the room temperature (21 °C). In Figure 6, an exemplary plot of Δ*T* vs. *t* for both bare and surface-modified samples is given. As a representative material, 316L steel was selected. As can be seen, surface modification of 316L steel results in a change in its temperature response observed during the friction test. The presented curves were recorded simultaneously with alternations in COF, shown in Figure 4A. First, it can be observed that small friction forces and the resultant COFs (Figure 4A, S3) correspond to low temperatures in the friction zone (Figure 6, S3). This is a typical phenomenon. Nevertheless, considering the data presented in Figure 4A and Figure 6, attention is drawn to the difference in the temperature response observed for bare 316L steel, as well as steel modified with TiN.

As described earlier, the average value of the friction coefficient recorded for the TiN-coated 316L steel was approx. 0.15 lower than that of the uncoated steel. However, the data presented in Figure 6 indicate that the mean Δ*T* of the WC-Co vs. TiN 316L pair was higher than that recorded for WC-Co vs. uncoated 316L. This phenomenon cannot be explained by the difference in the thermal conductivity of 316L steel and the TiN coating only. As indicated in the literature (Table 6), the thermal conductivity of the TiN coating is similar to that of 316L steel. The temperature measurement was carried out in a cemented carbide ball, WC-Co, with the highest thermal conductivity amongst all analyzed materials (Table 6). This would indicate that the friction pair which included steel modified with the TiN coating should be characterized by a similar temperature response as the uncoated sample. However, the recorded data, presented in Figure 6, show that, in a tribopair where TiN-coated 316L steel was used, the WC-Co ball tends to accumulate heat. Approximately 40 min after the start of the test, Δ*T* monitored for S2 (Figure 6) began to rise rapidly, reaching a maximum of 17.5 °C. However, after a few minutes of friction, the measured temperature of the tribological pair decreased to an average of 14.5 °C. On the contrary, the uncoated sample (S1) presented a temperature plateau at approx. 12 °C, with a slight downward trend. Therefore, it can be assumed that the observed temperature peak in sample S2 may have been caused by the damage of the TiN film, most probably its peeling off the metallic sample surface. However, this assumption needs to be confirmed in further tests. Nevertheless, the observed temperature phenomena prove that it is worth to monitoring counter sample temperature along with COF, as far more complex information on the current state of the tribological pair can be obtained. 

**Figure 7:**
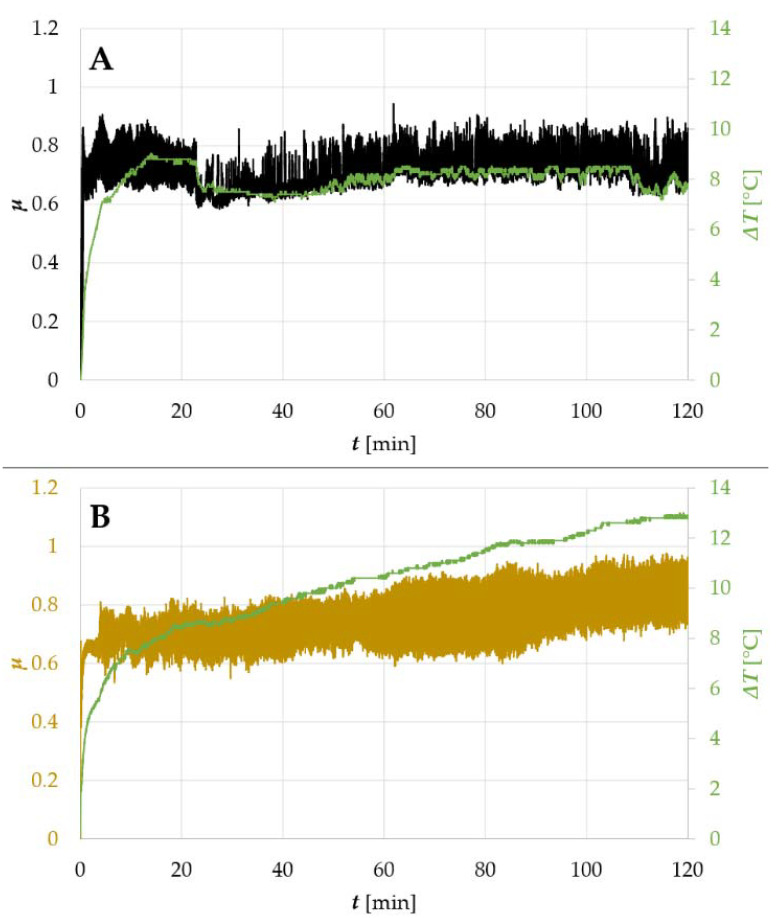
A comparison in frictional and thermal response of 440B stainless steel subjected to different surface finish: (**A**) uncoated sample (reference), (**B**) TiN-coated sample.

The described relationships between *t* and rapid increase in Δ*T*, followed by its subsequent stabilization, were observed also for titanium alloy Ti6Al4V. However, in TiN-coated 440B steel, the Δ*T* vs. *t* curve revealed a different trend (Figure 7B). For both 440B stainless steel specimens, bare and TiN-coated, the average COF remained at a similar level during the whole tribological test. Moreover, its oscillations were also similar (Figure 7A,B). Nevertheless, the temperature response, which was repeatable in all samples tested, followed different patterns. The Δ*T* was increasing in time, lacking stabilization. Compared with the uncoated sample (Figure 7A), the temperature response of the TiN-coated tribopair (Figure 7B) was much more pronounced, with an uptrend instead of a plateau.

**Figure 8:**
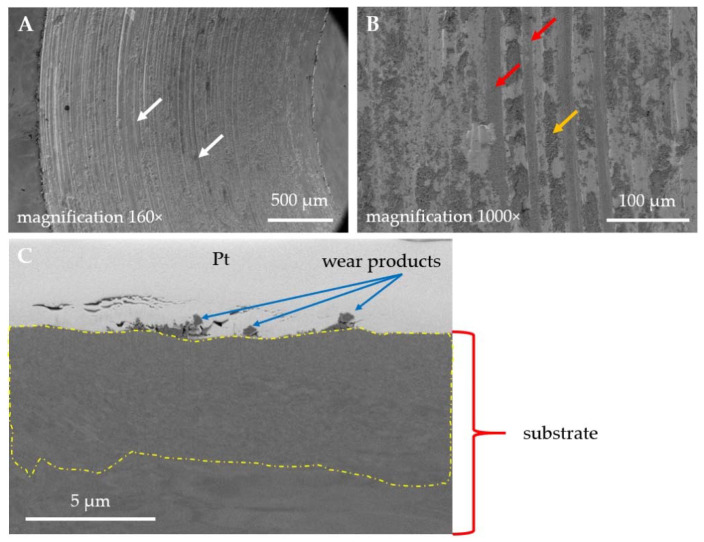
SEM micrographs of the wear tracks obtained for the uncoated 316L austenitic stainless steel (S1): (**A**,**B**) surface state of the wear track, (**C**) FIB-SEM cross-section of a wear track.

**Figure 9:**
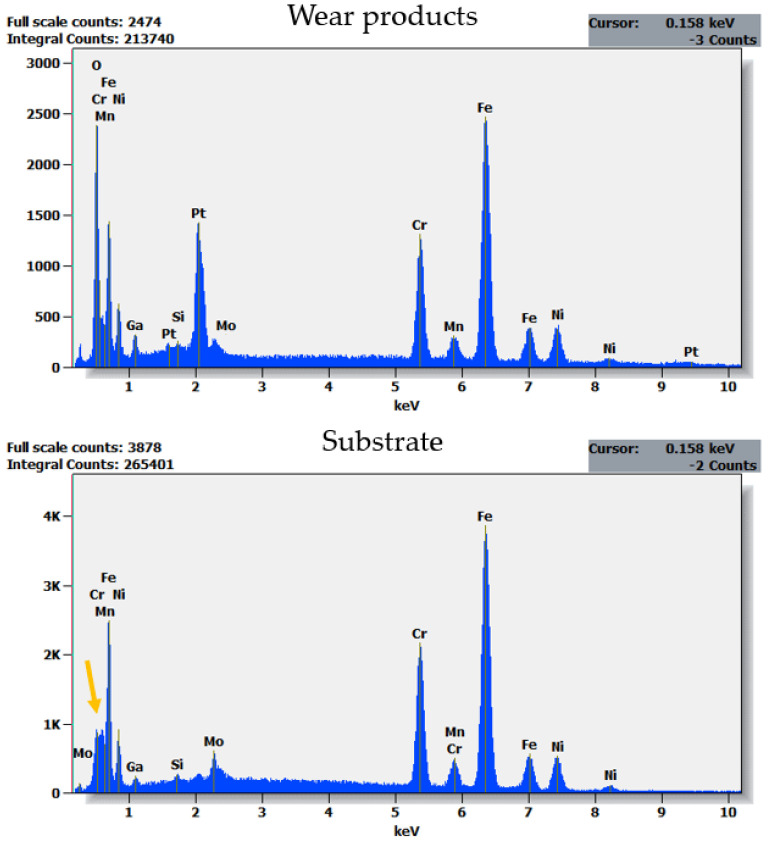
EDX spectra collected for a cross-section of the wear track obtained on the uncoated 316L stainless steel. The cross-section was obtained with the use of FIB. Therefore, Ga and Pt can be present in spectra. Note the oxygen lacking in spectra obtained for the substrate material (yellow arrow).

The observed differences in wear and frictional performance of the studied materials are reflected in findings from microscopic observations. In Figure 8, SEM micrographs of the wear track obtained for the uncoated 316L stainless steel are shown. The dominant wear mode is abrasive wear and ploughing (Figure 8A,B, red arrows), accompanied by plastic deformation of the substrate (Figure 8A, white arrows). The friction track is also covered with numerous wear products, marked with yellow arrows (Figure 8B). Moreover, a FIB-SEM cross-section was prepared on a wear track (Figure 8C). It was observed that the wear products are found only on the wear track surface and are not embedded in the substrate material. What is more, the substrate grains that are located directly below the wear track are smaller than the ones present ca. 5 µm below it (Figure 8, area showed with a yellow dashed line). However, further studies must be done to determine whether microstructure texturization takes place during friction. To check the elemental composition of the wear products, EDX analysis was done on a cross-section (Figure 8C). As the results of EDX analysis showed, the wear products which were found on the surface of the wear track are of an oxide nature (Figure 9). Therefore, the observed cross-dependence between the COF and the Δ*T* (Figure 4A and Figure 6) follows the previous literature reports. Odabas [49] showed that, in oxidizing steels, the COF and Δ*T* vs. time plots typically follow the same patterns.

**Figure 10:**
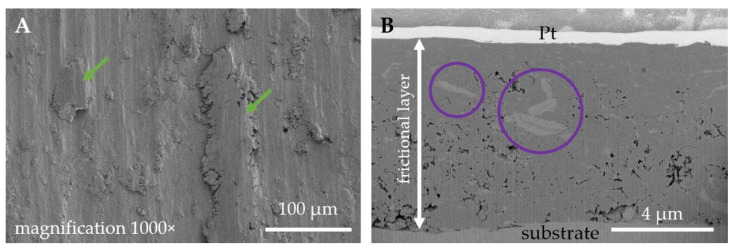
SEM micrographs of the wear tracks obtained for the TiN-coated 316L austenitic stainless steel (S2): (**A**) surface state of the wear track, (**B**) FIB-SEM cross-section of a wear track.

**Figure 11:**
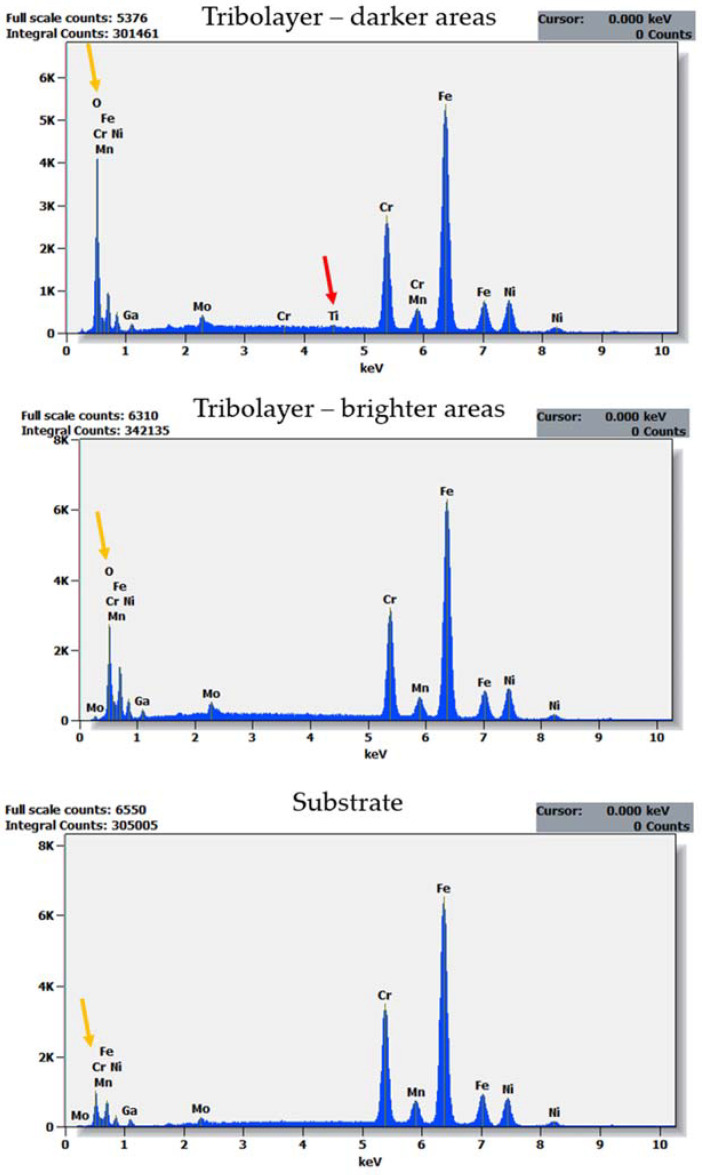
EDX spectra collected for a cross-section of the wear track obtained on the TiN-coated 316L stainless steel. The cross-section was obtained with the use of FIB. Therefore, Ga and Pt can be present in spectra. Note the oxygen lacking in spectrum obtained for the substrate material (yellow arrow), as well as very small amounts of Ti seen only in frictional layer (red arrow).

The dominant wear mode significantly changes when a coating is applied, contributing also to a difference in the thermal response of a counter sample (Figure 6). In Figure 10A, the wear track for TiN-coated 316L is presented. Compared with uncoated steel observed under the same magnification (Figure 8B), the number and size of free wear particles are substantially reduced. Plastic deformation of the substrate can be seen (Figure 10A, green arrows). Nevertheless, no remains of the TiN film can be found on the wear track surface. As SEM-FIB analysis showed, dominant wear mode changes into tribochemical wear, where a thick porous frictional layer is formed (Figure 10B). Such a layer was not observed in the uncoated steel (Figure 8C). As revealed by the EDX analysis (Figure 11), the thick tribolayer is formed from the oxidized wear products, mostly originated from the substrate material. In the porous frictional layer, a contrast difference is seen—in a dark matrix, some brighter areas can be found (Figure 10B, violet circles). According to the findings from the EDX study (Figure 11), the darker areas are richer in oxygen than the brighter ones. Moreover, only in the dark areas trace amounts of Ti, originated from the TiN coating, were found. In contrary to the uncoated steel (Figure 8C), as a result of wear, a thick frictional layer is formed instead of generating oxygen-rich free particles. Therefore, it can be stated that even if a coating is removed from the sample surface because of friction, it affects significantly the performance of a tribological pair. Here, a difference in the thermal response of tribological pairs was obtained (Figure 6). However, similarly to the uncoated steel, no signs of adhesive wear between the tested sample and the WC-Co ball were found, as no tungsten is present in the EDX spectra (Figure 11).

**Figure 12:**
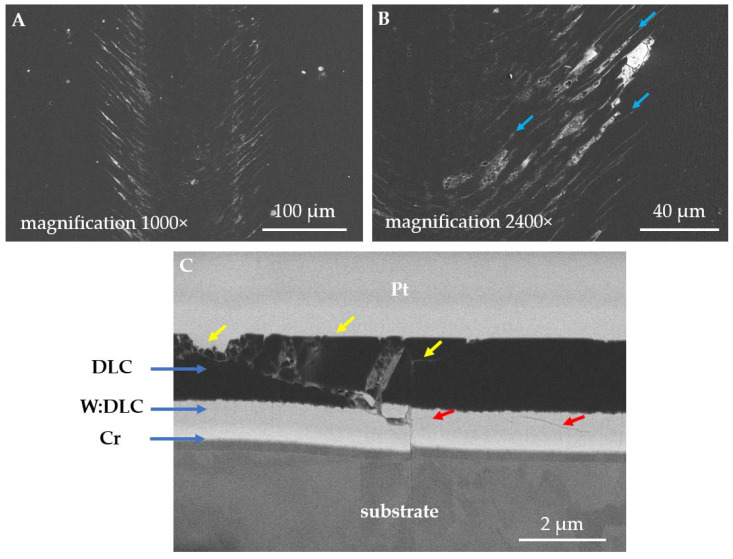
SEM micrographs of the wear tracks obtained for the DLC-coated 316L austenitic stainless steel (S3): (**A**,**B**) surface state of the wear track, (**C**) FIB-SEM cross-section of a wear track.

On the contrary, DLC film (Figure 12A) suffered mostly from localized damage. Compared with the uncoated or TiN-coated 316L steel (Figure 8B and Figure 10A), under the same magnification, only ca. 110 µm wide wear track is seen. It should be noted that the hairline-like cracks (Figure 12B, blue arrows) are arranged in the direction of sliding. The shape and position of cracks suggest that local damages of the film are a result of tensile stresses in the friction zone [50]. Moreover, local peeling of the DLC top layer is present at the tensile cracks (Figure 12B). The damage pattern is confirmed also in the SEM-FIB cross-sectional study (Figure 12C). The top DLC layer is severely cracked (Figure 12C, yellow arrows), and free abrasive particles are generated because of this process. Wear debris tends to fill the empty gaps between the cracks. Furthermore, severe cracking is present also at the DLC/W:DLC interface (Figure 12C, red arrows), which results in transferring particles of W:DLC in between the cracks of pure DLC. Nevertheless, the fatigue cracks did not propagate to the substrate material. However, the substrate suffered from plastic deformation.

**Figure 13:**
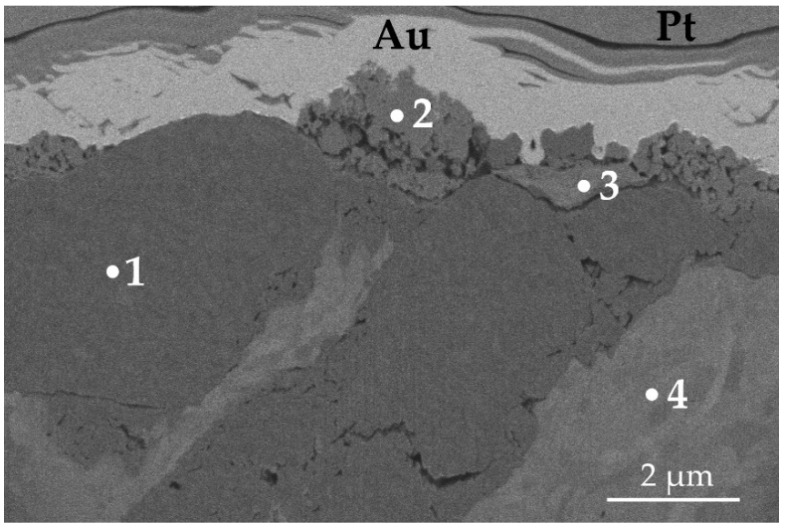
Backscattered electron SEM micrograph of the cross-section of a wear track, obtained on TiN-coated 440B stainless steel. The cross-section was done with the use of FIB. Au and Pt are the protective layers, used to prepare a cross-section with FIB.

**Figure 14:**
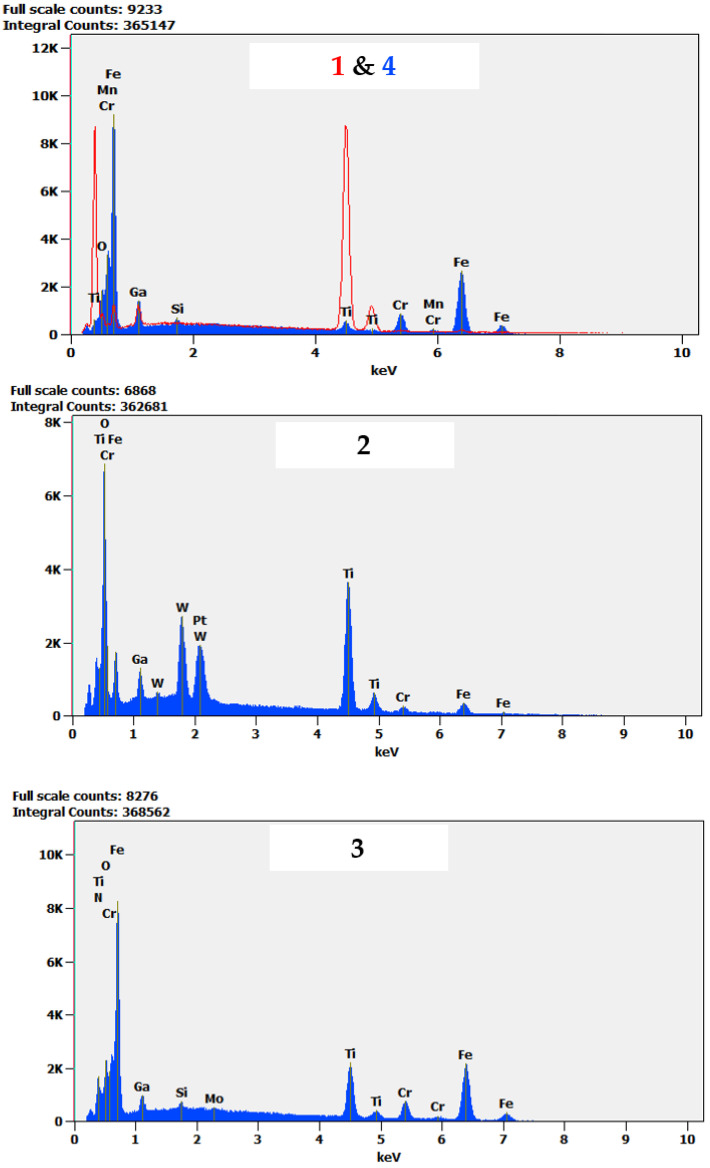
EDX spectra of areas: **1**–**4**, identified on a cross-section of a TiN 440B wear track presented in Figure 13. The cross-section was obtained with the use of FIB. Therefore, Ga is present in all spectra.

Moreover, a difference in dominant wear modes was observed when the substrate material was changed. In Figure 13, a cross-section of a wear track obtained on TiN-coated 440B stainless steel is presented. The cross-section of the wear track is characterized by a complex microstructure. First, the cross-sectional analysis confirms that the protective TiN film was severely damaged because of friction, as no intact coating can be seen (Figure 13). As the EDX analysis revealed, some areas of the cross-section fit the chemical profile of substrate material—440B stainless steel (Figure 13 and Figure 14, point no. 4). However, there are also dark, Ti-rich zones (Figure 13 and Figure 14, point no. 1). Using EDX spectra (Figure 14, spectrum no. 1) it can be concluded that those darker microconstituents also lack Fe, Cr, or Mn, which are the typical elements found in 440B steel. Therefore, it can be stated that during friction, particles of a damaged titanium nitride coating are being embedded in the substrate material. This assumption is confirmed by the lack of continuity between zones 1 and 4, as presented in Figure 13. Between both zones which significantly differ in chemical composition, microporosity is present (Figure 13).

Moreover, in Figure 13, on top of Ti-rich region, two porous phases, denoted as “2” and “3”, are present. Compared with zones 1 and 4, a significant difference in EDX spectra of both areas 2 and 3 is seen. First, tungsten is seen only in EDX spectrum no. 2 (Figure 14). The only possibility for W to appear in the friction track was the material transfer from the counter sample, the WC-Co ball. While only solitary regions rich in W are detected, it can be stated W-rich microsized wear products were embedded in the friction layer. Considering high oscillations of COF in tribological pair with TiN-coated 440B, it can be concluded that W-rich particles were transferred because of adhesive wear. Moreover, a high amount of oxygen was detected in both spectra 2 and 3 (Figure 14). Therefore, it can be concluded that in frictional contact, a new tribolayer was formed out of oxygen absorbed from the environment, as well as the wear products originating from both counter body and tested sample. Mostly this oxygen- and iron-rich tribolayer is seen at the surface of the wear track, presented in Figure 13.

**Figure 15:**
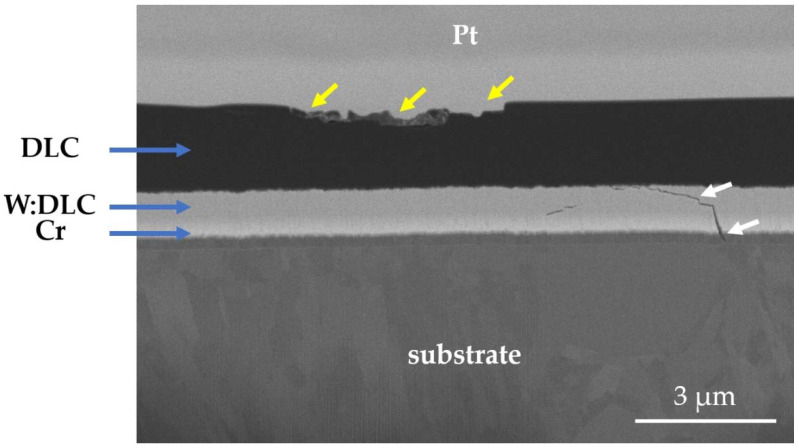
Backscattered electron SEM micrograph of the cross-section of a wear track, obtained on DLC-coated 440B stainless steel. The cross-section was obtained with the use of FIB.

However, once again, the dominant wear modes change significantly if a hybrid DLC film is deposited on a 440B steel surface instead of TiN. In Figure 15, the cross-section of a wear track obtained on DLC-coated 440B stainless steel is presented. According to the findings, after 2 h of friction, DLC coating becomes only locally damaged. The wear of the material is concentrated mainly in the top DLC layer (Figure 15, yellow arrows). However, fatigue cracking of the support W:DLC layer is present (Figure 15, white arrows). Moreover, the Cr adhesive layer which lays on top of the substrate has also been affected. The crack propagates to substrate material as well (Figure 15, white arrows).

**Figure 16:**
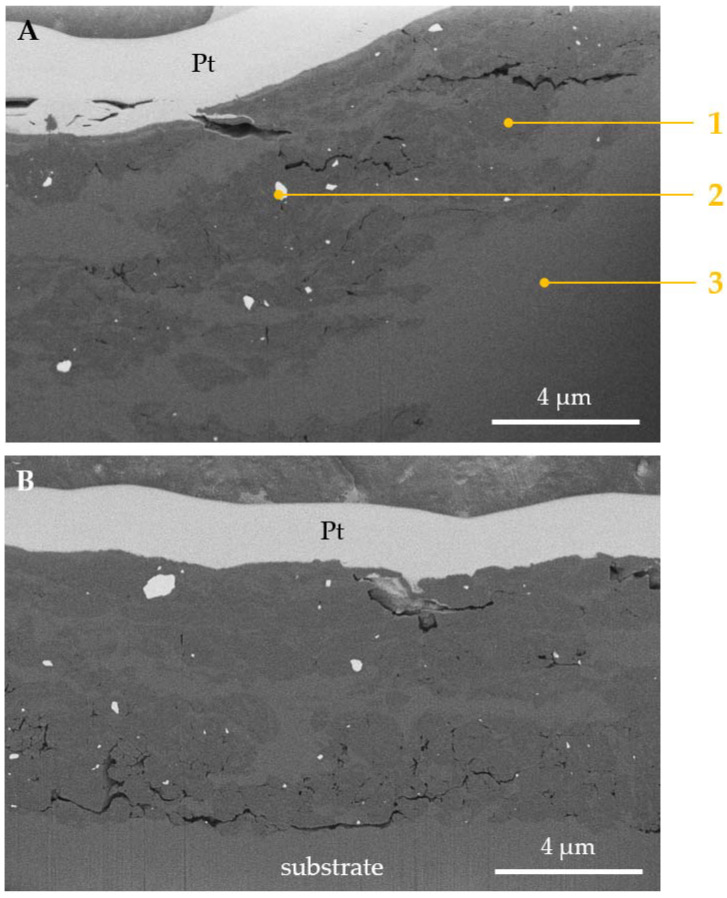
Backscattered electron SEM micrographs of the cross-section of a wear track, obtained on: (**A**) uncoated Ti6Al4V alloy, (**B**) TiN-coated Ti6Al4V alloy. The cross-sections were prepared with the use of FIB.

**Figure 17:**
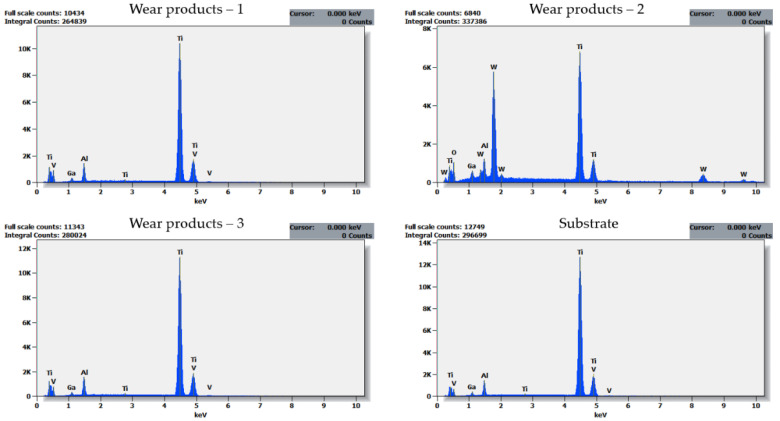
EDX spectra of areas: **1**–**3** identified on a cross-section of Ti6Al4V wear track presented in Figure 16A. The cross-section was obtained with the use of FIB. Therefore, Ga is present in all spectra. For comparison purposes, spectrum of a pure substrate material was added.

For Ti6Al4V, entirely different wear modes were observed. In Figure 16, cross-sections of the wear tracks obtained on both: uncoated (Figure 16A) and TiN-coated (Figure 16B) titanium alloy are shown. As can be seen, in both samples tested, a complex microstructure of the wear track is present. Dark grey, light grey, as well as almost white microconstituents can be seen in both uncoated and TiN-coated samples (Figure 16A,B). For these microconstituents, EDX analysis was performed (Figure 16A and Figure 17). According to the results of the EDX analysis, the elemental composition of areas 1 and 3 is similar. Moreover, it does not differ much from the composition of a pure substrate (Figure 17). Therefore, it can be stated that compared to both tested stainless steels, the tribolayer is not formed out of the oxidized wear products. However, in Ti samples, one more micro constituent which was not present in steels is seen. This new micro constituent denoted as “2” in Figure 16A, is rich in tungsten. Based on the W presence, as well as dispersion of the W-rich particles in the frictional layer, it can be concluded that adhesive transfer between the WC-Co counter body and the tested titanium samples occurred. However, while the frictional layers seem similar, some differences can also be distinguished. As presented in Figure 16A,B, the thickness of the wear-affected zone is reduced when a TiN coating is applied. Due to that, as mentioned before, the difference in thermal response between the uncoated and TiN-coated sample is seen.

**Figure 18:**
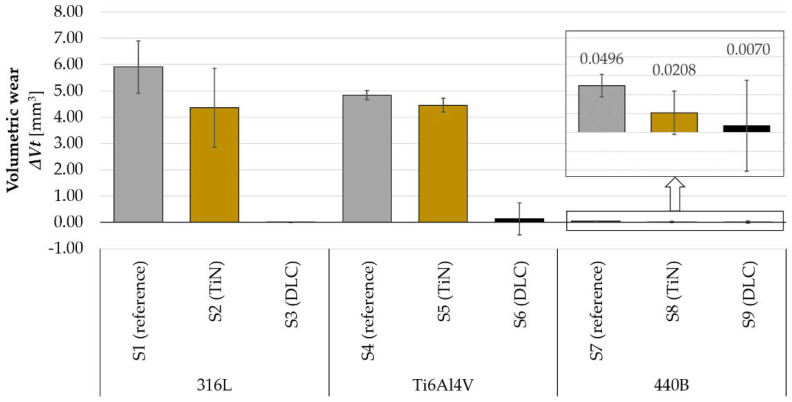
Volumetric wear Δ*V_t_* of the tested samples, *n* = 5.

After the frictional tests, the volumetric wear of all samples was measured. Findings from these measurements are presented in Figure 18. As can be seen, the 440B steel is characterized by the lowest volumetric wear amongst all substrates tested, regardless of the applied surface modification method. While 440B martensitic stainless steel is harder than 316L or Ti gr. 5 (Table 5), these results are of no surprise. However, only in the case of the Ti6Al4V titanium alloy, the application of a protective TiN coating resulted in a reduction in wear of the substrate material (Figure 18). For both steels (316L and 440B), in comparison with their bare references, TiN coating had no statistically significant effect on volumetric wear. On the other hand, DLC did not have a significant effect on wear performance only in 440B stainless steel.

## 4. Discussion

As presented in the study, both substrate materials and the applied coatings substantially affect the tribological response of the frictional pair. Compared with the available literature data, some of the observed phenomena provide new insight into the friction and wear processes of coated metals. As presented in this study, in surface-modified tribopairs, there is no simple relationship between COF, temperature response, dominant wear mode, and volumetric wear of the tribological pair. In Figure 6, the Δ*T* of samples: S1, S2, and S3, is presented. The rapid temperature increase in the friction zone was detected only in the TiN-coated sample (Figure 6, S2). While it is known that TiN coating did not survive the frictional test (Figure 10), this behavior can be explained by the peeling off and wear of the protective coating. It is possible that the observed temperature peak (Figure 6, S2) corresponds to the rapid degradation of TiN and the subsequent crushing of wear debris in the contact zone. In the work by De Oliveira Junior [51], it was stated that the relative hardness of the abrasive particles in relation to the material surfaces in contact significantly affects the wear rate. The greater the hardness ratio is, the more severe abrasive wear is seen [51]. While TiN is relatively harder than both stainless steels and the counter sample [19], it can be concluded that its detached particles affected the wear regime. Mild wear transformed into severe abrasion [51]. The hard particles, present in the friction zone, acted as an abrasive medium. In turn, the processes of severe abrasive wear directly affected the heat level in the contact zone [52]. However, as friction progressed, the TiN particles were transformed into fine debris, which eventually was removed from the friction zone. Therefore, a drop in Δ*T* was observed.

Using this example, it is worth stressing the importance of temperature sensing in the friction zone or its proximity. Based on data presented in Figure 4A only, it is not possible to determine if the TiN coating becomes severely damaged during friction. However, considering the data provided in Figure 6, one may suspect that the thin film may not have survived the frictional test. Nevertheless, this assumption needs to be confirmed with the use of microscopy (Figure 10). Moreover, a tendency to maintain, in relation to the uncoated samples, the increased temperature in the contact zone until the end of the test, was observed for both TiN coated steels—AISI 440B and 316L (Figure 6 and Figure 7). This phenomenon was not seen in bare steels (Figure 6 and Figure 7).

Using 440B stainless steel as an example, it was revealed that as a result of friction, the TiN coating was destroyed and its particles were embedded below the oxygen-rich tribolayer (Figure 13 and Figure 14). Formation of the oxygen- and iron-rich tribolayers on TiN-coated steels was discussed also by Saravanan et al. [53], as well as Wilson and Alpas [54]. According to Saravanan et al. [53], a tribolayer is formed at a later stage of friction, when heavy deformation of the substrate occurs with the accompanying temperature rise in the friction zone. This assumption would comply with the results presented in this paper. In the work by Straffelini et al. [17], it was suggested that oxygen- and iron-rich friction layer may present insulating properties. This could explain the tendency of the counter sample to maintain higher temperatures until the end of the test in comparison to the uncoated steels (Figure 6 and Figure 7). On the contrary, the friction layer which is formed at the surface of both 316L and 440B stainless steels, did not reveal the tendency to promote the accumulation of heat in the friction zone (Figure 6 and Figure 7). This is associated with the different nature of the tribolayer, in which chromium oxides are prevalent. While the Cr- and O-rich friction layer is being worn by each revolution of the sample, the stainless-steel surface is tribochemically polished. Therefore, a friction track as presented in Figure 7A is observed.

However, different wear regimes were observed in DLC-coated samples (Figure 12 and Figure 15). In DLC films, more than 50% of bonds reveal sp^2^ hybridization [23], which is typical for graphite [55]. This way, superior antifriction properties of the coating are obtained. Solis et al. [2] observed that in dry sliding conditions, the heat phenomena that occur in the friction zone contribute to hydrogen depletion in the DLC matrix, leading to its transformation from diamond-like to the graphite-like structure. Therefore, low friction forces are observed (Figure 4). Nevertheless, it was revealed that the DLC coating suffered from damage induced by the tensile stresses (Figure 12). This led to angular cracking of the film and the subsequent damage of the top layer of the hybrid coating (Figure 12), which corresponds to local damages seen on the sample surface. Despite this, in all substrates tested, DLC-coated samples revealed the smallest wear, friction forces, and Δ*T* in tribological pair with WC-Co.

In numerous works, it is stressed that tribological coatings should be selected in accordance with the physical and mechanical properties of the underlying substrate. For example, for TiN and TiAlN coatings, the importance of matching Young’s modulus of the substrate material and the film was reported [3]. However, using as an example two stainless steels (316L and 440B) of similar Young’s moduli, we revealed that the frictional performance of a coated tribosystem may differ drastically (Figure 18). The wear phenomena that occur when the coating becomes damaged to result in a lack of advantage that is being sought in surface modification. In our study, we have shown that under selected sliding conditions, only Ti gr. 5 benefited from TiN film in terms of reducing the volumetric wear of the material (Figure 18).

While our experiment is limited by the test rig, it provides new knowledge to the phenomena of friction, tribofilms formation, as well as frictional heating in dry sliding conditions. Though the thermocouple was installed approx. 1 mm from the friction zone, the choice of a hard WC-Co pin ensured repeatability of the experiments. As wear of WC-Co balls was negligible, the constant height of the thermocouple was maintained throughout the whole tribological test. Therefore, even though the exact temperature in the friction zone was not measured, it was possible to observe thermal trends and tendencies of the frictional pair. For example, we have observed that the damage of a protective coating may contribute to excessive friction heating through modification of the dominant wear modes of the tribopair.

## 5. Conclusions

In this paper, the frictional behavior of surface-coated 316L austenitic stainless steel, Ti6Al4V two-phase titanium alloy, and 440B martensitic stainless steel was discussed. For comparison purposes, specimens were subjected to surface modification with titanium nitride or a hybrid diamond-like carbon coating. As a reference, bare samples were used. During the tribological test, both changes in the friction force, as well as the counter sample temperature, were monitored. Moreover, volumetric wear was measured for each tribopair. According to the results provided, the following conclusions can be drawn:

In all substrate materials tested, compared with the uncoated samples, under applied testing conditions, titanium nitride coating contributed to greater frictional heating of a tribopair. Though in all samples tested it was revealed that the TiN film did not survive the frictional test, its presence favored the formation of thick frictional layers which adversely affected the heat transfer from the friction zone. In stainless steels, oxygen-rich films were formed. In titanium alloy, a frictional layer consisting of the substrate and counter-sample material was formed.

In some tribopairs tested, it was revealed that temperature monitoring can provide information on whether the coating was severely damaged. A temperature peak, most probably corresponding to the failure of the protective film, was seen, e.g., in TiN-coated 316L steel. However, further studies on this topic are required.

## Figures and Tables

**Table 1:** Chemical composition of AISI 316L austenitic stainless steel (wt.%), adapted from ref. [33].

Cr	Ni	Mo	C	Si	Mn	P	S	Fe
16.7	12.6	2.1	<0.03	<0.6	1.6	<0.03	<0.03	balance

**Table 2:** Chemical composition of Ti6Al4V two-phase titanium alloy (wt.%), adapted from ref. [34].

Al	V	C	O	Ti
6.0	3.9	0.03	0.15	balance

**Table 3:** Chemical composition of AISI 440B martensitic stainless steel (wt%), adapted from ref. [35].

C	Cr	Mo	Si	Mn	Fe
0.95 ÷ 1.20	16.0 ÷ 18.0	0.75	1.0	1.0	balance

**Table 4:** Vickers hardness of substrate materials used in the study [36,37,38,39].

316L	Ti6Al4V	440B
195	320 ÷ 360	770

**Table 5:** Summary of all samples tested. The number of Ra measurement replications in each series was equal to 10.

Series No.	Disc Material	Surface Modification	Ra (µm)	Number of Samples Tested
S1	316L	none (uncoated)	0.015 ± 0.002	5
S2		TiN	0.021 ± 0.004	5
S3		DLC	0.017 ± 0.002	5
S4	Ti6Al4V	none (uncoated)	0.024 ± 0.003	5
S5		TiN	0.026 ± 0.004	5
S6		DLC	0.024 ± 0.002	5
S7	440B	none (uncoated)	0.017 ± 0.001	5
S8		TiN	0.023 ± 0.003	5
S9		DLC	0.018 ± 0.002	5

**Table 6:** Thermal conductivity of the materials subjected to friction tests [41,42,43,44,45,46,47,48].

Material	Thermal Conductivity (W/mK)
316L	15.0
TiAl4V	6.6 ÷ 6.7
440B	15.0
WC-Co	36.0 ÷ 6 0.0
TiN	11.0 ÷ 19.0
DLC	0.8 ÷ 1.0

## Data Availability

The data presented in this study are available on request from the corresponding author.

## Nomenclature

CAE	cathodic arc deposition,
CVD	chemical vapor deposition,
Δ*T*	the temperature of each friction pair (°C), understood as: Δ*T* = *T_a_* − *T_p_*, where *T_a_* is the temperature of the friction pair recorded during the wear tests, and *T_p_* is the room temperature (21 °C),
Δ*V_t_*	volumetric wear of discs (mm^3^),
DLC	diamond-like-carbon,
EDX	energy dispersive X-ray spectroscopy,
F	normal load (10 N),
FIB	focused ion beam,
MS	magnetron sputtering,
*µ*, COF	coefficient of friction,
PACVD	plasma assisted chemical vapor deposition,
PVD	physical vapor deposition,
Ra	the arithmetic average of the absolute values of the profile height deviations from the mean line (µm),
SEM	scanning electron microscopy,
*T*	friction force (N),
TiN	titanium nitride.
